# Supplementary material for: How to normalize metatranscriptomic count data for differential expression analysis
Source: PeerJ. 2017 Oct 17;5:e3859. doi: 10.7717/peerj.3859 (PMC5649605; doi:10.7717/peerj.3859)
Supplement: File S4 [file peerj-05-3859-s004.pdf]

Supplemental material for "How to normalize metatranscriptomic count data for differential expression analysis"

H. Klingenberg and P. Meinicke
University of Göttingen

July 21, 2017

Table 1: Species names and associated abbreviations for real metatranscriptome data

Abbreviation	species name
BACCAC	*B. caccae ATCC 43185*
BACOVA	*B. ovatus ATCC 8483*
BACUNI	*B. uniformis ATCC 8492*
BDI	*P. distasonis ATCC 8503*
BT	*B. thetaiotaomicron VPI-5482*
BVU	*B. vulgatus ATCC 8482*
%	*B. cellulosilyticus WH2*
CLOSCI	*C. scindens ATCC 35704*
CLOSPI	*C. spiroforme DSM 1552*
COLAER	*C. aerofaciens ATCC 25986*
%	*D. longicatena DSM 13814*
RUMOBE	*R. obeum ATCC 29174*

Abbreviations and species names for the organisms observed in the real metatranscriptome data [1]. For *B. cellulosilyticus WH2* "%" indicates that this organism does not appear in the comparison of "day 13" vs "day 27" and therefore an abbreviation is not required. Note that this organism could not be mapped to the genes required for the combined Pfam feature vector.

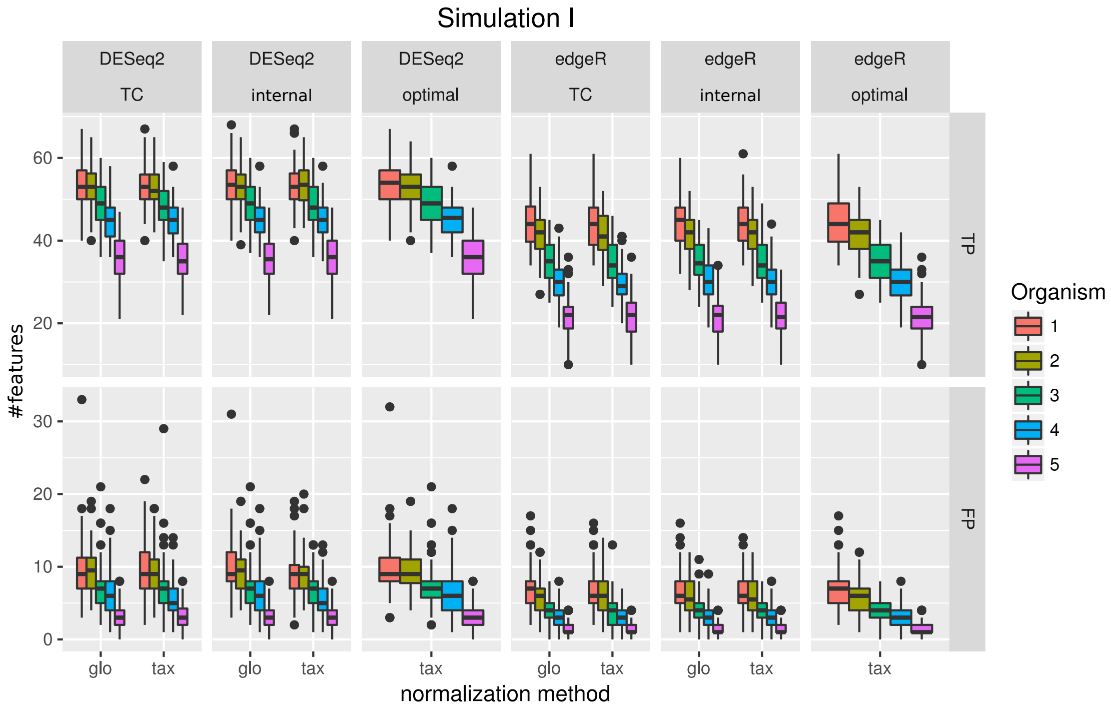

Figure 1: Boxplots based on the number of true positives (TP) and false positives (FP) for 5 organisms with library sizes according to Simulation II parameters. Here, the random factor for LS variation is from a reduced interval between 0.75 and 1.25. Performance for global (glo) and taxon-specific (tax) scaling over 100 runs of the simulation. For the analysis DESeq2 and edgeR were used in combination with three different normalization methods: “TC” refers to total count normalization, “internal” indicates the internal normalization implemented in DESeq2/edgeR and “optimal” uses the optimal scaling factors for taxon-specific scaling.

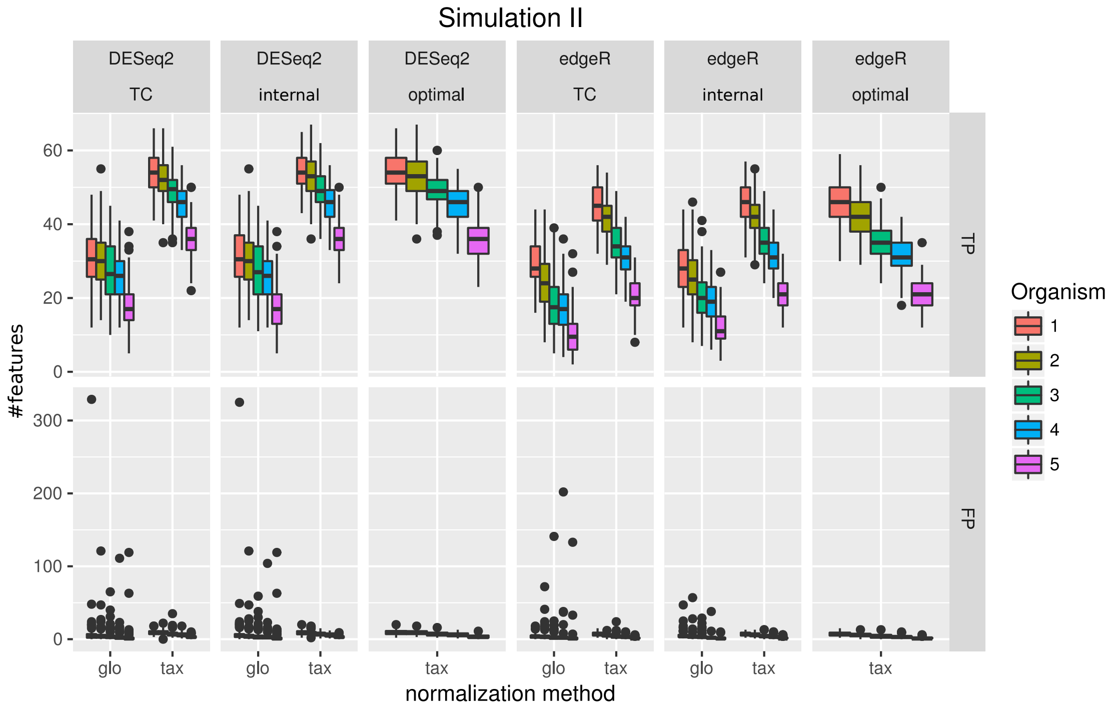

Figure 2: Boxplots based on the number of true positives (TP) and false positives (FP) for 5 organisms with library sizes according to Simulation II parameters for global (glo) and taxon-specific (tax) scaling over 100 runs of the simulation. For the analysis DESeq2 and edgeR were used in combination with three different normalization methods: "TC" refers to total count normalization, "internal" indicates the internal normalization implemented in DESeq2/edgeR and "optimal" uses the optimal scaling factors for taxon-specific scaling.

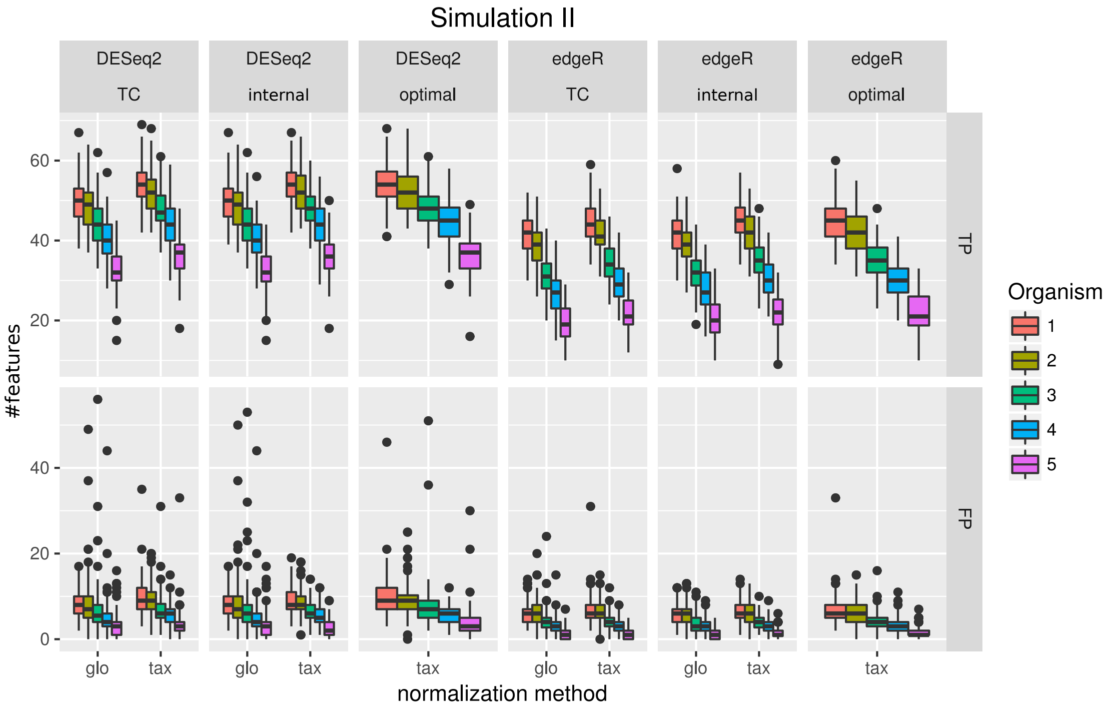

Figure 3: Boxplots based on the number of true positives (TP) and false positives (FP) for 5 organisms with library sizes according to Simulation II parameters. Here the random factor for LS variation is from a reduced interval between 0.75 and 1.25. For the analysis DESeq2 and edgeR were used in combination with three different normalization methods: "TC" refers to total count normalization, "internal" indicates the internal normalization implemented in DESeq2/edgeR and "optimal" uses the optimal scaling factors for taxon-specific scaling.

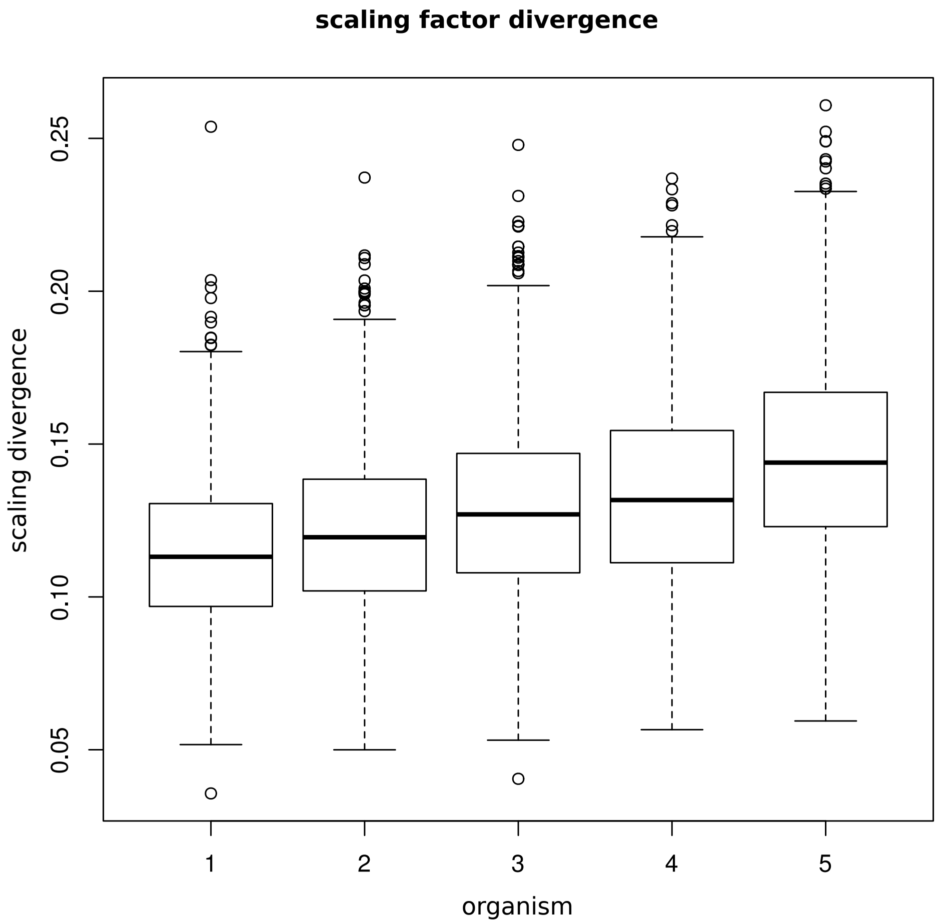

Figure 4: Boxplots of the scaling divergence for global scaling over 1000 iterations of simulation II. The organisms are shown on the x-axis, the library size (base count) is the highest for organism “1” and the lowest for organism “5”.

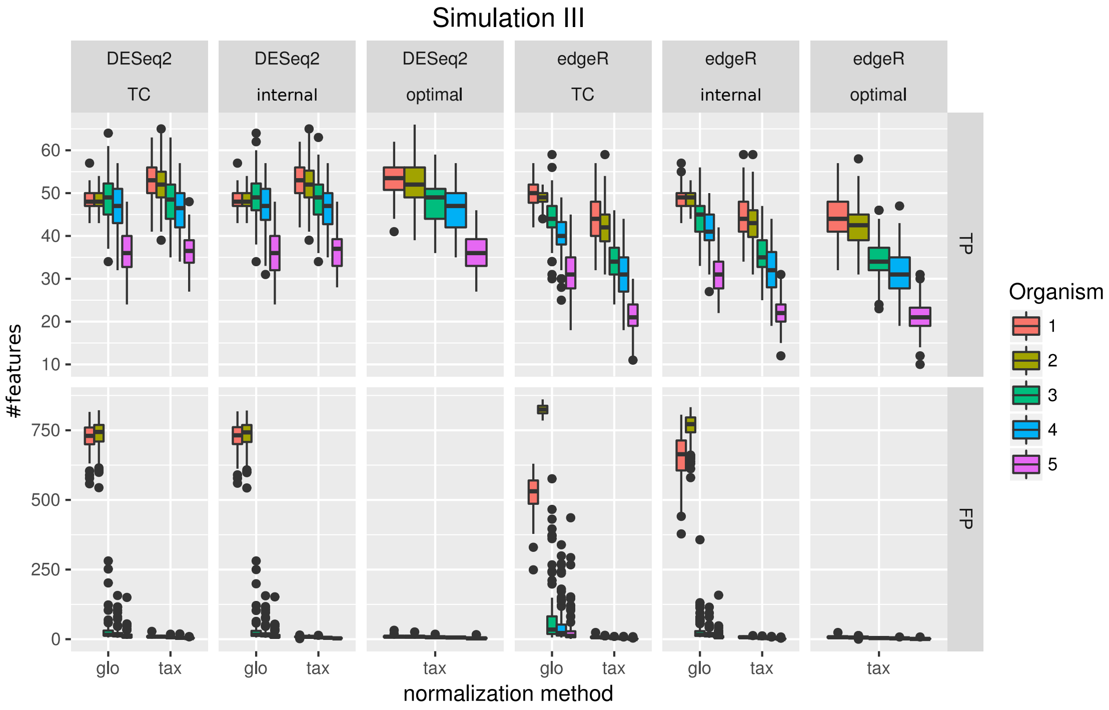

Figure 5: Boxplots based on the number of true positives (TP) and false positives (FP) for 5 organisms with library sizes according to simulation III parameters for global (glo) and taxon-specific (tax) scaling over 100 runs of the simulation. For the analysis DESeq2 and edgeR were used in combination with three different normalization methods: "TC" refers to total count normalization, "internal" indicates the internal normalization implemented in DESeq2/edgeR and "optimal" uses the optimal scaling factors for taxon-specific scaling.

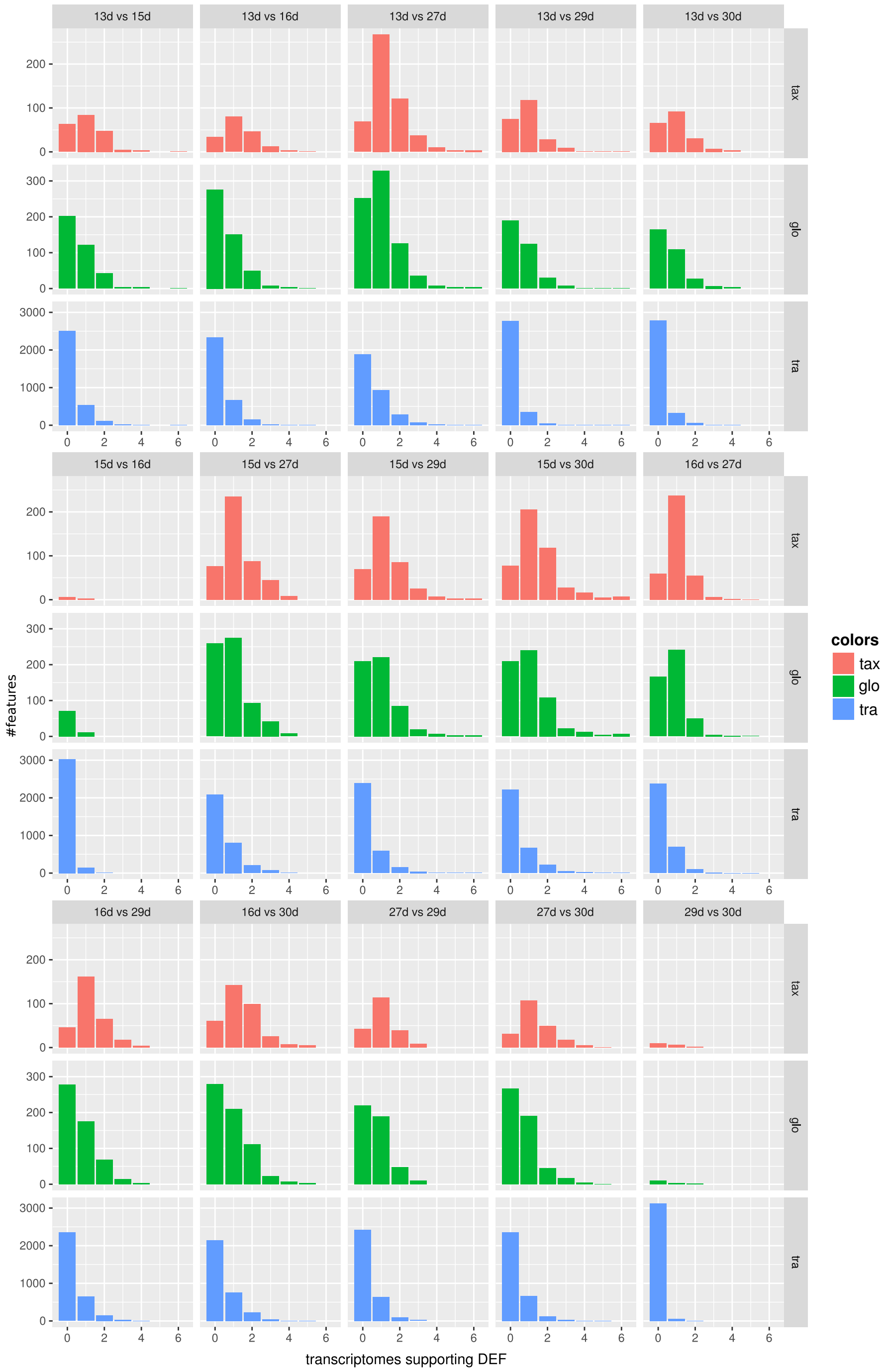

Figure 6: Histograms for predicted DEF in real data according to the number of single organism analyses that show a significant difference (x-axis) for global scaling ("glo", green) and taxon-specific scaling ("tax", red). The histogram for ("tra", blue) displays the number of features identified as DE and the number of supporting organisms for the single transcriptomes.

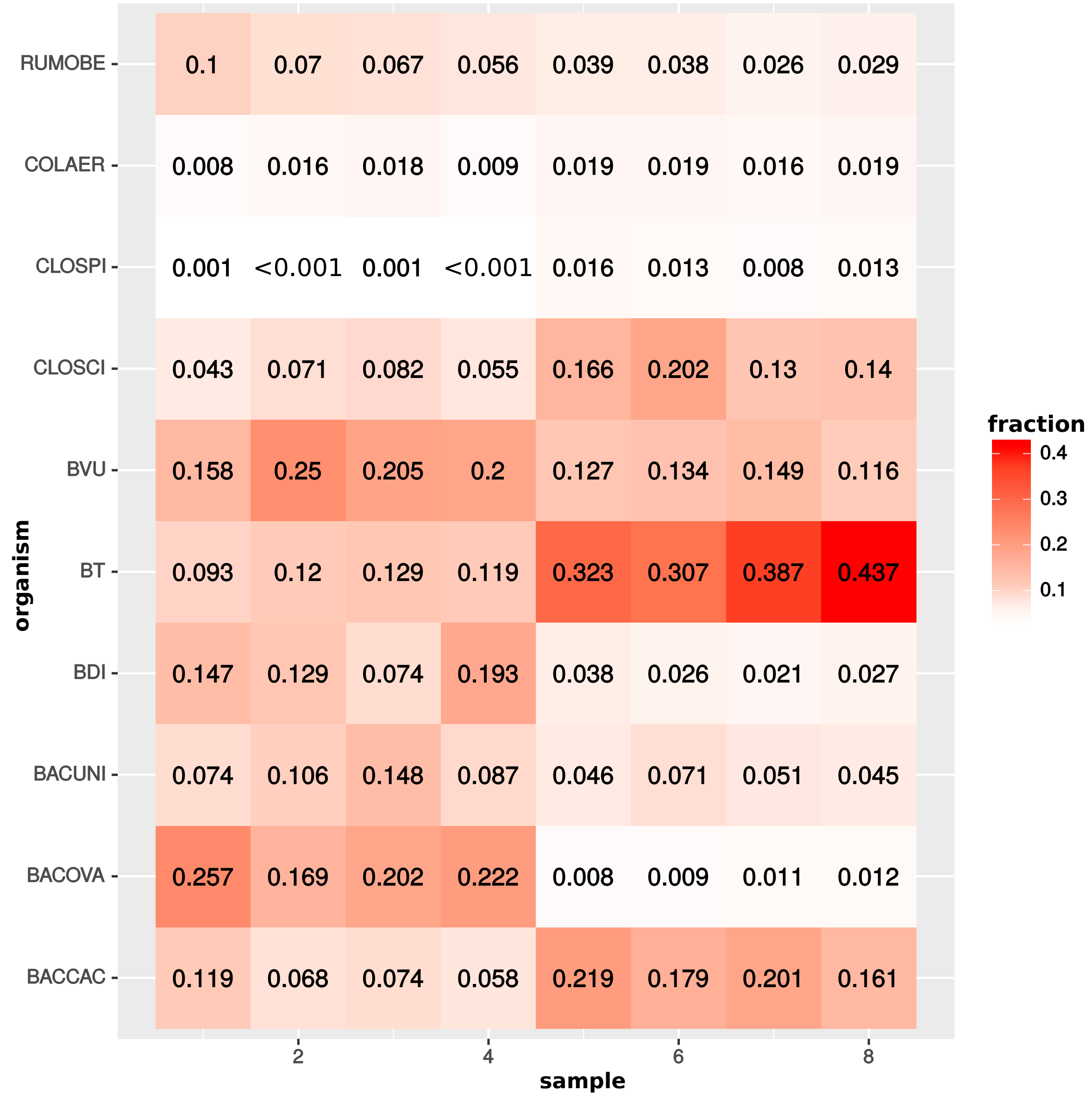

Figure 7: Sample-specific fractions of organisms in comparison “day 13” vs “day 27”. Samples 1-4 correspond to condition “day 13” and 5-8 correspond to condition “day 27”. For the species name abbreviations see Additional File 2: Tab. 1.

References

[1] McNulty, N.P., Wu, M., Erickson, A.R., Pan, C., Erickson, B.K., Martens, E.C., Pudlo, N.A., Muegge, B.D., Henrissat, B., Hettich, R.L., Gordon, J.I.: Effects of diet on resource utilization by a model human gut microbiota containing *Bacteroides cellulosilyticus* wh2, a symbiont with an extensive glycobiome. PLoS Biol **11**(8), 1–20 (2013). doi:10.1371/journal.pbio.1001637
